# DNA polymeraseη protein expression predicts treatment response and survival of metastatic gastric adenocarcinoma patients treated with oxaliplatin-based chemotherapy

**DOI:** 10.1186/1479-5876-8-126

**Published:** 2010-11-27

**Authors:** Kai-yuan Teng, Miao-zhen Qiu, Zhuang-hua Li, Hui-yan Luo, Zhao-lei Zeng, Rong-zhen Luo, Hui-zhong Zhang, Zhi-qiang Wang, Yu-hong Li, Rui-hua Xu

**Affiliations:** 1 State Key Laboratory of Oncology in South China, Guangzhou 510060, China; 2 Department of Medical Oncology, Sun Yat-Sen University Cancer Center, Guangzhou 510060, China; 3 Department of Pathology, Sun Yat-Sen University Cancer Center, Guangzhou 510060, China

## Abstract

**Background:**

DNA polymerase η (pol η) is capable of bypassing DNA adducts produced by cisplatin or oxaliplatin and is associated with cellular tolerance to platinum. Previous studies showed that defective pol η resulted in enhanced cisplatin or oxaliplatin sensitivity in some cell lines. The purpose of the present study was to investigate the role of pol η protein expression in metastatic gastric adenocarcinoma.

**Methods:**

Four gastric adenocarcinoma cell lines were chosen to explore the relationship between pol η protein expression and oxaliplatin sensitivity by western blotting and MTT assay. Eighty metastatic gastric adenocarcinoma patients treated with FOLFOX or XELOX regimen as first-line chemotherapy were analyzed, corresponding pretreatment formalin-fixed paraffin-embedded tumor tissues were used to detect pol η protein expression by immunohistochemistry. Relationship between pol η protein expression and clinical features and outcome of these patients was analyzed.

**Results:**

A positive linear relationship between pol η protein expression and 48 h IC50 values of oxaliplatin in four gastric cancer cell lines was observed. Positivity of pol η protein expression was strongly associated with poor treatment response, as well as shorter survival at both univariate (8 versus 14 months; P < 0.001) and multivariate (hazard ratio, 4.555; 95% confidence interval, 2.461-8.429; P < 0.001) analysis in eighty metastatic gastric adenocarcinoma patients.

**Conclusions:**

Our study indicates that polη is a predictive factor of treatment response and survival of metastatic gastric adenocarcinoma patients treated with FOLFOX or XELOX as first-line chemotherapy. Therefore confirming the value of polη in studies with prospective design is mandatory.

## Background

Stomach cancer is the fourth most common cancer worldwide, with 603,003 new cases among men and 330,290 new cases among women per year [1]. It is the second most common cause of cancer related death (700,000 deaths annually), with almost two-thirds of the cases occurring in developing countries and 42% in China alone [2]. Surgery remains the major potential method to cure the disease; however, approximately 84% of gastric cancer patients will develop to be an advanced disease, with 30% of locally advanced cases, 30% metastatic diseases at diagnosis, and 24% recurrence diseases [3]. The literatures showed that the median survival was only 3-4 months among advanced gastric cancer patients without chemotherapy. The new generation of chemotherapeutic agents, such as Oxaliplatin, can prolong survival in advanced gastric cancer to be 10 to 12 months; moreover, chemotherapy can also improve the quality of life [4-12]. Therapeutic effect mainly depends on the response of drugs to tumor during the first-line chemotherapy, because so far only one small phase III study with 120 cases showed a modest survival benefit from irinotecan monotherapy over supportive care alone [13]. Unfortunately, due to drug resistance, only 30-50% response rates can be achieved even though administrating new generation drugs such as docetaxel, oxaliplatin, capecitabine, irinotecan, S1, etc to advanced gastric cancer patients as first-line treatment [3]. That means at least 50% patients have to undergo ineffective treatment, which may not only decrease the patients' quality of life, but also increase the economic burden. So how to predict response of chemotherapy agents in gastric cancer is a very important scientific issue.

Oxaliplatin is the third generation platinum, playing a vital role in chemotherapy for gastrointestinal cancer. Oxaliplatin-based combination regimen such as oxaliplatin plus 5-FU or 5-FU-like drug has been proven to be active in about 40-50% of advanced gastric cancer patients [14-16]. Oxaliplatin and cisplatin share the similar mechanism, and cause mono-adducts and intra-strand or inter-strand cross-links in the double DNA helix that severely block DNA synthesis [17-19]. When this happens, some pathways of DNA damage repair may switch on, including nucleotide excision repair (NER), mismatch repair (MR), homologous recombination (HR), translesion DNA synthesis (TLS) [20]. These adduct repairs occur primarily through NER [21]. TLS is another alternative way to repair these lesions, which is mainly done by DNA polymerases η[22]. Polymeraseη(Polη), one of lesion-replicating enzymes, incorporates the correct nucleotide over lesions such as a platinum adduct by TLS and continues chain elongation, whereas classical pols cannot [23]. Polη has the highest efficacy of bypassing Pt-GG intra-strand diadducts caused by platinum among these lesion-replicating enzymes, with limited fidelity [23,24]. Recent experiments have shown that the absence of polη results in a statistically significant enhancement in cisplatin sensitivity when comparing polη-null Xeroderma Pigmentosum-variant human fibroblasts with polη-expressing ones [25]. This enhancement is also observed when the cells were treated with carboplatin and oxaliplatin [25]. Recent data show that polη mRNA level negatively correlated with cisplatin sensitivity of non small cell lung cancer (NSCLC) cell lines [26].

In the present study, we report for the first time the relationship between polη protein expression and oxaliplatin sensitivity of gastric cancer cell lines and the significance of that in predicting treatment response and survival of metastatic gastric cancer patients treated with oxaliplatin-based chemotherapy.

## Materials and methods

### Cell lines

The gastric cancer cell lines, including SGC7901, AGS, MKN45, and MGC803, were donated by Professor Libing Song from State Key Laboratory of Oncology in Southern China (Cancer Center of Sun yet-sen University). All cell lines were maintained in RPMI 1640 (Gibco) supplemented with 10% fetal bovine serum (Gibco), except MKN 45 with 20% fetal bovine serum.

### Patients and Samples

Patients in our clinical database with chemotherapy-naïve, histologically proven metastatic advanced gastric cancer were enrolled for the study. All patients had to receive FOLFOX (fluorouracil, leucovorin and oxaliplatin) or XELOX (capecitabine and oxaliplatin) regimen as first-line chemotherapy at Cancer Center of Sun Yat-sen University, and formalin-fixed paraffin-embedded pretreatment samples under gastroscope biopsies or palliative operation were obtained. Histopathologic characteristics were confirmed by blinded review of the original pathology slides. The TNM classification was used for pathologic staging, and the World Health Organization classification was used for pathologic grading. Other inclusion criteria included age between 18-80, Eastern Cooperative Oncology Group (ECOG) performance status of 2 or less, second line chemotherapy or not, no radiation treatment. All patients provided written informed consent; we obtained separate consent for use of specimens. Study approval was obtained from independent ethics committees at Cancer Center of Sun Yat-Sen University. The study was undertaken in accordance with the ethical standards of the World Medical Association Declaration of Helsinki.

### Follow-up and evaluations

Patients were followed up by telephone or letter communication once every year for a total of 4 years. Overall survival was defined as the time from the date of confirmed diagnosis to death and censored at the date of last contact for a surviving patient. Disease response was evaluated according to the Response Evaluation Criteria in Solid Tumours (RECIST criteria) [27].

### Cytotoxicity assays

Cell growth inhibition was determined by 3- (4,5-dimethylthiazol-2-yl) -2,5-diphenyltetrazolium bromide assay (MTT assay). Briefly, cells were seeded in 96-well plates and allowed to attach overnight. After 48 hours of drug incubation at various concentrations (37°C), MTT reagent (5 mg/mL, 20 μL/well) was added to each well and incubated for an additional 4 hours. The plates were then centrifuged (1,500 g, 5 minutes) and the supernatant was removed. The cell pellets were dissolved in 200 uL DMSO. Absorbance was determined using the Model 550 Microplate Reader (BIO-RAD, Hercules, CA, USA) at a wavelength of 570 nm, with background subtraction at a wavelength of 630 nm. All experiments were performed in triplicate. The concentration required to inhibit cell growth by 50% (IC50) was calculated from survival curves using the Bliss method [28].

### Western blotting

Cells were washed with ice-cold phosphate buffer saline and harvested in sampling buffer [62.5 mmol/L Tris-HCl (pH 6.8), 2% SDS, 10% glycerol, and 5% 2-h-mercaptoethanol]. Protein concentration was determined by Bradford assay (Bio-Rad Laboratories). Equal amounts of proteins were applied to 7.5% polyacrylamide SDS gels (SDS-PAGE), separated electrophoretically, and transferred onto polyvinylidene fluoride membranes. After blocked in 5% non-fat milk in TBST buffer (10 mmol/L Tris-HCL, 150 mmol/L NaCl, and 0.1% Tween20, pH 8.0) for 1 h at room temperature, the membrane was incubated with anti-polη rabbit antibody (1:400; Abcam). Polη expression was detected with horseradish peroxidase-conjugated goat anti-rabbit IgG and enhanced chemiluminescence. Anti-α-tubulin antibody was used as the loading control.

ImageJ software from National Institutes of Health (NIH) was employed to quantify protein.

### Immunohistochemistry

Immunohistochemical (IHC) analysis was done to detect polηprotein expression in 80 human gastric cancer tissues. Briefly, the tissue sections were deparaffinized in xylene at 37°C for 20 minutes and rehydrated. Endogenous peroxide was blocked by incubating the sections with 3% hydrogen peroxide in methanol for 20 minutes at 37°C. Then the sections were submerged into 10 mM citrate buffer (pH 6.0) and microwaved for antigenic retrieval, followed by incubation with rabbit anti- polη (1:100; Abcam) overnight at 4°C. After washing, tissue sections were treated with horseradish peroxidase-labeled secondary antibody for 30 minutes. The sections were developed with diaminobenzidine tetrahydrochloride (DAB) and counterstained with hematoxylin.

Analysis of immunohistochemistry in our study was carried out by two independent observers based on the proportion of positively stained tumor cells. If there is difference between these two observers, these slides were reinvestigated by both investigators using a multiheaded microscope. Tumors with more than 5% of POLη-positive cancer cells were regarded as positive (nucleus staining), otherwise negative.

### Statistical analysis

Receiver operating characteristic (ROC) curve analysis and Fisher's exact test were performed to select IHC Pol-positive value with highest accuracy. 2 × 2 table was constructed yielding sensitivity, specificity, positive and negative predictive value, and accuracy was calculated as proportion of true positive and true negative patients out of whole patients. All statistical analyses were performed by SPSS 15.0 statistical software package (SPSS Inc, Chicago, IL, USA). P value < 0.05 was considered to be statistically significant. Kaplan-Meier analysis with log-rank testing was used for univariate analyses. Variables showing a trend for association with survival (P < 0.05) were selected for inclusion in the final multivariate Cox proportional hazards model. The relationship between polη expression and clinicopathologic characteristics was examined by a chi-square test and Fisher's exact tests.

## Results

### Polη expression correlates with oxaliplatin sensitivity of gastric cancer cell lines

**Figure 1:**
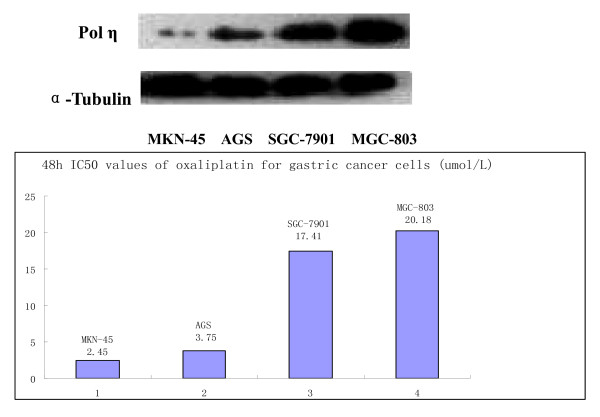
**Expression analysis of POL η protein in gastric cancer cell lines by western blotting and 48 h IC50 values of oxaliplatin for gastric cancer cells (umol/L)**.

**Figure 2:**
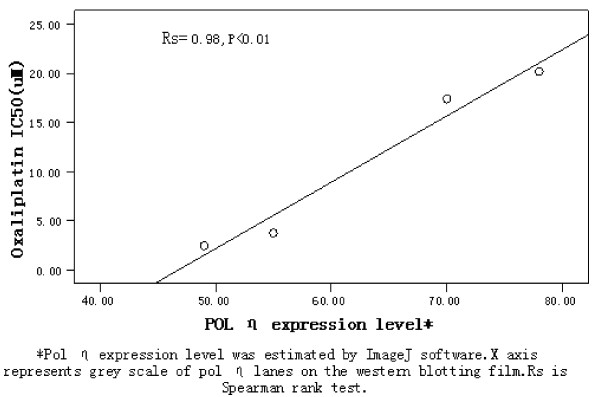
**Correlation between POL η expression and IC50 of oxaliplatin for gastric cells**.

Oxaliplatin sensitivity of four gastric cell lines (SGC7901, AGS, MKN45, and MGC803) were detected by MTT assay described above. Endogenous polη protein expression of four cell lines were compared with each other by western blotting and the half maximal inhibitory concentration (IC50) values of oxaliplatin for cells were shown in Figure 1. Significant and positive correlation was observed, as shown in Figure 2 when compared polη protein expression with IC50 values of oxaliplatin.

### Patient characteristics

Eighty patients from January 2005 and July 2009 were retrospectively analyzed. Patients had a median age of 54 (range, 26.0-79.0), with 49 males and 31 females. Other clinical characteristics were summarized in Table 1. The response rate (CR+PR) with first-line XELOX or FOLFOX chemotherapy was 47.5%, clinical benefit rate (CR+PR+SD) was 77.5%. Part of these patients (23/80) had second-line chemotherapy.

**Table 1:** Patient characteristics (N = 80)

characteristic	No. of patients	%
Age(yrs)		
Median	54.0	
Range	26.0-79.0	
Sex		
Male	49	61.3
Female	31	38.7
ECOG performance status		
0	36	45.0
1	42	52.5
2	2	2.5
Primary sites		
Cardia	21	26.3
Body	18	22.5
Antrum/pylorus	41	51.2
Metastatic sites		
Liver	26	32.5
Lung	21	26.3
Peritoneum	19	23.8
Others	14	17.4
Pathologic differentiation N0		
G1	2	2.5
G2	26	32.5
G3	52	65.0
Treatment Response (1st line)		
CR	2	2.5
PR	36	45.0
SD	24	30.0
PD	18	22.5
2^nd^-line chemotherary regimen		
BSC	57	71.3
FOLFIRI	6	7.5
XELIRI	8	10.0
DX	6	7.5
TP	3	3.7

CR, complete response; PR, partial response; SD, stable disease; PD, progression disease;

BSC, best supportive care; FOLFIRI, 5-fluracil plus leucovorin plus irinotecan; XELIRI, capecitabine plus irinotecan; DX, docitaxel plus capecitabine; TP, paclitaxel plus cisplatin.

### The criteria that tumor tissue with more than 5% of Polη-positive cancer cells was defined as IHC-positive has highest accuracy in predicting clinical benefit of first line chemotherapy

**Figure 3:**
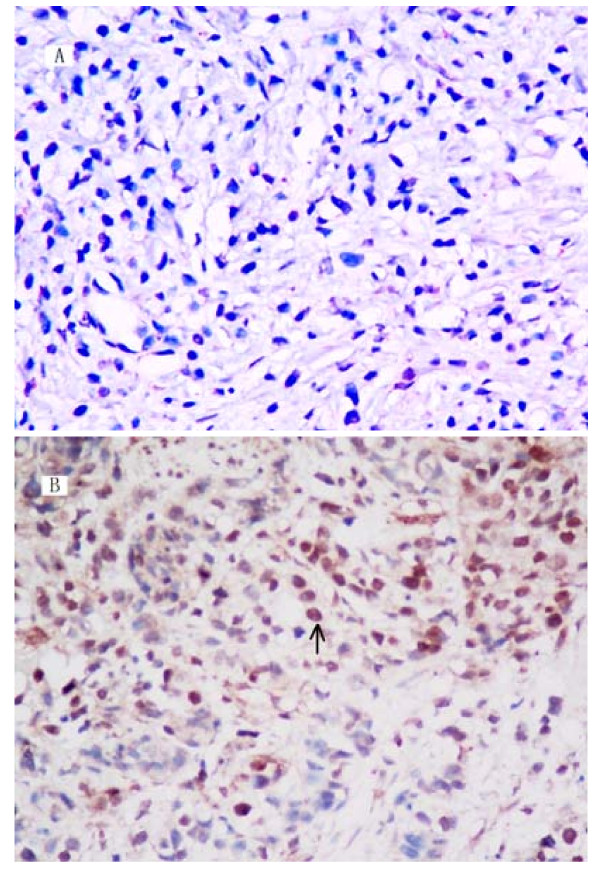
**The expression of POLη protein in advanced gastric cancer as examined by immunohistochemistry**. A. negative expression image in tumor tissue(× 400). B. POLη protein was detectable in the nucleus of gastric cancer cell (× 400).

**Figure 4:**
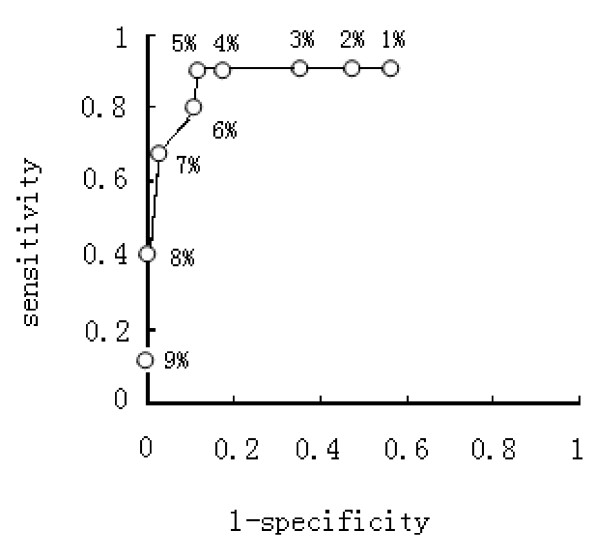
**Receiver operating characteristic curve of Polη IHC counting in predicting chemotherapy response to FOLFOX or XELOX regimen**.

Tumor tissues of eighty metastatic gastric cancer patients treated with FOLFOX or XELOX regimen were used to detect polη protein expression by Immunohistochemistry (Figure 3). Because the percentage of nucleus-staining tumor cells in all cases was no more than 10%, we tried to select IHC Polη-positive value with highest accuracy to predict clinical benefit of first line chemotherapy. We defined nine IHC-positive value: ≥ 1%, ≥ 2%, ≥ 3%, ≥ 4%, ≥ 5%, ≥ 6%, ≥ 7%, ≥ 8%, ≥ 9%, and constructed nine 2× 2 table. Table 2 is showed as an example. Sensitivity, specificity, positive and negative predictive value, and accuracy was calculated and used to draw ROC curve. As showed in Table 3 and Figure 4 cut off value ≥ 5%, has highest accuracy (88.8%). So tumors with more than 5% of POLη-positive cancer cells were regarded as positive (nucleus staining), otherwise negative.

**Table 2:** DNA polymerase η protein expression and clinical treatment response

	Clinical failure	Clinical benefit	Total cases
Pol η (+)	16	7	23
Pol η (-)	2	55	57
Total cases	18	62	80

≥ 5% Polη expression in tumor cells defined as positive.

**Table 3:** Accuracy, sensitivity, specificity, positive predictive value, and negative predictive value according to Polη IHC counting percent in predicting chemotherapy response to FOLFOX or XELOX regimen

Tumor cell positive percent, cut off	Specificity	Sensitivity	PPV	NPV	Accuracy
1%	0.419	0.889	0.308	0.929	0.525
2%	0.500	0.889	0.340	0.940	0.588
3%	0.645	0.889	0.421	0.952	0.700
4%	0.823	0.889	0.593	0.962	0.838
5%	0.887	0.889	0.696	0.965	0.888
6%	0.887	0.778	0.667	0.932	0.863
7%	0.978	0.667	0.705	0.904	0.863
8%	0	0.389	1.000	0.849	0.863
9%	0	0.111	1.000	0.795	0.800

PPV, positive predictive value; NPV, negative predictive value.

### Relationship of polη expression with the clinical features of metastatic gastric cancer

As shown in Table 4 Only 23 of 80 cases (28.75%) had polη protein positive expression (≥ 5% Polη-positive cancer cells), 16 of the 23 positive cases (69.56%) failed to the treatment, in contrast, only 2 of the 57 negative cases (3.51%) had progressed disease after FOLFOX or XELOX chemotherapy. Polη expression strongly correlated with treatment response to oxaliplatin-based chemotherapy of metastatic gastric cancer (P < 0.001), whereas it is not associated with age, gender, primary sites, metastatic sites or pathologic differentiation levels.

**Table 4:** Correlation between POL η expression and clinicopathologic characteristics of gastric cancer patients

characteristic	POLη		χ^2 ^test P value	Fisher's test P value
			
	Positive (n = 23)	Negative (n = 57)		
Age(y)				
< 60	16	37	0.690	0.797
≥ 60	7	20		
Gender				
Male	14	35	0.695	1.000
Female	9	22		
Primary sites				
Cardia	10	11	0.084	0.102
Body	4	14		
Antrum/pylorus	9	32		
Metastatic sites				
Liver	11	15	0.247	0.269
Lung	5	16		
Peritoneum	3	16		
Others	4	10		
Pathologic differentiation				
G1	2	0	0.068	0.107
G2	6	20		
G3	15	37		
Treatment Response				
CR+PR+SD	7	55	< 0.001	< 0.001
PD	16	2		

CR, complete response; PR, partial response; SD, stable disease; PD, progression disease.

Spearman correlation analysis was further done to confirm the correlation between polη expression and clinicopathologic features. Pearson contingency coefficient shown that polηexpression levels was significantly related with treatment response (P < 0.001), likewise, no significant correlations with other factors was obtained (data not shown).

### Relationship between polη expression and survival of metastatic gastric cancer

**Figure 5:**
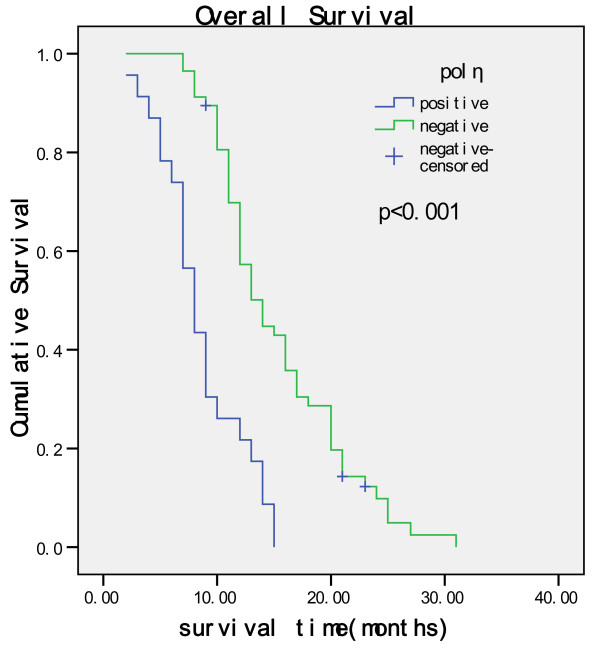
**Kaplan-Meier curves with univariate analysis (log-rank test) for patients with negative POLη expression versus positive POLη expression tumors**.

Kaplan-Meier univariate survival analysis revealed that the positive expression of polη in tumor cells and poor treatment response were significantly associated with shorter survival. The median survival time for polη positive and negative cases were 8 and 14 months respectively, as shown in Figure 5 (log rank, P < 0.001). Multivariate survival analysis (Cox regression model) revealed that the expression of polη was independent prognostic factors. The 95.0% confidence interval (CI) for relative risk was 2.461-8.429 (Table 5). These results indicated that the expression of polη in tumor tissue predicted shorter survival. No relationship was observed between the survival and the rest clinicopathological parameters such as age, gender, primary tumor sites, pathologic differentiation and metastatic sites.

**Table 5:** Multivariate analysis of overall survival in gastric carcinoma

Factors	Characteristics		Hazard ratio	95%CI	P value
				
	Unfavorable	Favorable			
Age	≥ 60	< 60	0.956	0.570-1.602	0.863
Histological grade	Poorly	Well/moderately	1.428	0.861-2.369	0.168
POL η	positive	negative	4.555	2.461-8.429	< 0.001

CI, confidence interval.

## Discussion

The present study partially revealed the role of DNA polymeraseη as a DNA repair protein in gastric cancer by detecting its expression in four gastric cancer cell lines and 80 patients with metastatic gastric adenocarcinoma who had received FOLFOX or XELOX as the first line chemotherapy. The results showed that the expression level of polη in tumor cell lines was correlated with the sensitivity of oxaliplatin (Figure 2). For the tumor tissue, the positive expression only occurred in 28.75% cases (23 out of 80), and the expression was modest. However, strong correlation was found between polη expression and treatment response as well as survival. All the results demonstrated that polη positivity was an indicator for poor treatment response and shorter survival in patients of above settings.

DNA polymeraseη is coded by POLH gene which is one of the 150 human DNA repair genes, whose defection results in Xeroderma Pigmentosum Variant (XP-V) syndrome, manifesting highly sensitivity to UV radiation and a trend to develop skin cancer [29-31]. Polη is an important lesion-replicating enzyme that replicates across pyrimidine dimers introduced by UV radiation, avoiding high gene mutation [32]. In addition to pyrimidine dimers, polη has been shown to replicate across cisplatin cross-linked intrastrand GG sites [33]. Some researches showed that polη expression was correlated with sensitivity to cisplatin or oxaliplatin in XP-V human fibroblasts cell and lung cell lines, and polη seemed to be a treatment-response predictive marker in NSCLC patients with cisplatin-based chemotherapy [26,27].

Less is known about the role of polη in gastric cancer. It is the first study to detect the sensitivity of oxaliplatin in these four gastric carcinoma cell lines (SGC7901, AGS, MKN45, and MGC803) by MTT assay and protein expression by western blotting. We found a significant linear relationship between them. Our study was to some extent consistent with the observation in lung cancer cell lines by Paolo Ceppi et al, though what they detected were polη mRNA level and cisplatin sensitivity [27].

Then we restrospectively analyzed the expression of polη protein in eighty metastatic advanced gastric cancer patients who received FOLFOX or XELOX as chemotherapy. We firstly observed that the percentage of polη-staining tumor cells in all 80 cases was no more than 10%, so we defined ≥5% as IHC positive according to the accuracy in predicting clinical benefit with XELOX or FOLFOX chemotherapy. With this standard, only 23 patients had positive expression, with 7 out of 62 clinical benefit cases (CR+PR+SD) from chemotherapy and 16 out of 18 PD cases. The expression rate between clinical benefit group (7/62, 11.3%) and PD group (16/18, 88.9%) was significantly different (P < 0.001). This indicated that Polη positivity might predict ineffective chemotherapy with XELOX or FOLFOX regimen. The result was consistent with our study in cells that Polη expression negatively correlates with oxaliplatin sensitivity of gastric cancer cell lines.

We found a significant survival benefit in polη negative patients. This benefit probably came from effective therapy of oxaliplatin, given that polη was strongly correlated with treatment response. It is well known that effective chemotherapy would enhance life quality of gastric cancer patients and improve survival; our investigation can help to predict the treatment response of oxaliplatin-based chemotherapy and survival, hence avoid unnecessary treatment at the beginning.

There are several limitations to our study. First, This is a retrospective study with a small number of patients. In the preset study, no significant association between polη expression and age, gender, primary tumor sites, metastatic sites or pathologic differentiation was obtained on the basis of such cases number. It is possible to get significant results in some clinicopathologic characteristics such as primary sites or pathologic differentiation (P value close to 0.05), if with enough patients. Therefore enlargeing the case number and performing prospective trials is mandatory. Second, because the test accuracy was calculated using the criteria defined by ROC curve analysis, our results needed validation in an independent cohort. Third, the expression of polη is relatively low in gastric cancer, due to subjectivity, the error of IHC counting could be bigger, which may influence the sensitivity and specificity when predicting treatment response, so more accurate method is needed. Forth, It is well known that platinum resistance is very complicated, so polη, as a single parameter, is hard to predict therapy response in an exact manner.

In spite of limitations, our study is one of the few attempts to define criteria for in vivo chemosensitivity of oxaliplatin-containing regimen using clinical response as reference standard. It may be an effective and cost-saving method to predict treatment response.

## Conclusion

In conclusion, the present study demonstrated that polη is a significant predictor of treatment response in patients with metastatic gastric cancer receiving chemotherapy of FOLFOX or XELOX. Such a marker might help clinicians to choose the optimal clinical strategy for patients with advanced gastric cancer. Moreover, the multivariate survival analysis revealed that the expression of polη was independent prognostic factors. The findings need to be confirmed in a larger prospective trial before application in clinical practice.

## Abbreviations

GG: guanine-guanine; CR: complete response; DNA: deoxyribonucleic acid; FOLFOX: fluorouracil, leucovorin and oxaliplatin; HR: homologous Recombination; mRNA; messenger ribonucleic acid; IC50: the half inhibitory concentration; IHC: immunohistochemistry; MMR: mismatch Repair; WB: western blotting; MTT assay: 3- (4,5-dimethylthiazol-2-yl) -2,5-diphenyltetrazolium bromide assay; NER: nucleotide Excision Repair; OS: overall survival; PD: progression disease; Pol η: polymerase eta; PR: partial response; SD: stable disease; Pt: platinum; ROC: receiver operating characteristic; TLS: translesion synthesis; XELOX: capecitabine and oxaliplatin; XPV: xeroderma pigmentosum variant.

## Competing interests

We have no financial or personal relationships with other people or organizations that would bias our work. No benefits in any form have been received or will be received from a commercial party related directly or indirectly to the subject of our article.

## Authors' contributions

KYT carried out the cytotoxicity assay and the IHC, participated in the clinical data collecting of the gastric carcinoma patients and drafted the manuscript. MZQ participated in the clinical data collecting and drafted the manuscript. ZHL carried out the cytotoxicity assay. HYL performed the statistical analysis. ZLZ participated in the design of the study. RZL and HZZ reviewed the IHC slices. ZQW and YHL participated in the statistical analysis. RHX conceived of the study, and participated in its design and coordination and helped to draft the manuscript. All authors read and approved the final manuscript.
